# Supplementary material for: Content-rich biological network constructed by mining PubMed abstracts
Source: BMC Bioinformatics. 2004 Oct 8;5:147. doi: 10.1186/1471-2105-5-147 (PMC528731; doi:10.1186/1471-2105-5-147)
Supplement: Additional File 2 — The original results of the above study (non-essential files are deleted to keep the file size under the limit set by BMC bioinformatics). [file 1471-2105-5-147-S2.bz2 › chilibotAdditionalFile2/dip10/204ID8915609E697/html/APP_TTR.html]

 


 **APP** and **TTR** 
  
Found 113 abstracts in PubMed, retrieved 10.  
 

 What does Google say? 
 PDF only 
| .edu only 

---

**Interactive relationship** (e.g. stimulation, inhibition, etc)

**Neutral relationship**- Taken together, these results support a model by which p CIP functions as a ligand dependent adapter, through specific protein protein interactions with AD1  [ **APP** ] , to recruit members from at least two distinct families of acetyltransferase  [ **TTR** ]  proteins to NRs.  Ref: 12885766 J Biol ChemJ Biol Chem,
- **TTR**  is an amyloid beta  [ **APP** ]  protein scavenger, so an increase in its expression could prevent amyloid aggregate formation.  Ref: 12566565 Proc Natl Acad Sci U S A, 2003
- Cultured muscle fibers CMF from a patient with inclusion body myositis IBM and cardiac amyloidosis associated with the transthyretin  [ **TTR** ]   **TTR**  Val122Ile mutation contained aspects of the IBM phenotype vacuolation, congophilic inclusions, and clusters of immunocolocalizing amyloid beta  [ **APP** ]  peptide Abeta and  **TTR**  accumulations.  Ref: 12874414 Neurology, 2003

**Non-interactive relationship** (e.g. studied together, co-existance, homology, etc.)

- Our data suggest that the choroid plexus cells produce molecules involved in processes such as prevention of fibrillization of amyloid beta  [ **APP** ]  protein transthyretin  [ **TTR** ]  and gelsolin, lipid metabolism phospholipid transfer protein and ABCA8, and detoxification androgen inducible aldehyde reductase.  Ref: 12614681 Neuroscience, 2003
